# Assessing acceptance and feasibility of a conversational agent to support individuals living with post-traumatic stress disorder

**DOI:** 10.1177/20552076241286133

**Published:** 2024-10-07

**Authors:** Hee Jeong Han, Sanjana Mendu, Beth K Jaworski, Jason E Owen, Saeed Abdullah

**Affiliations:** 1 8082 Pennsylvania State University, University Park, PA, USA; 2 2511 National Institutes of Health, Sacramento, CA, USA; 3 130477 National Center for PTSD, Menlo Park, CA, USA

**Keywords:** Conversational agent, chatbot, mental health, PTSD, self-management, supporting system

## Abstract

**Objective:**

Post-traumatic stress disorder (PTSD) is a pervasive health concern affecting millions of individuals. However, there remain significant barriers to providing resources and addressing the needs of individuals living with PTSD. To address this treatment gap, we have collaborated with clinical experts to develop PTSDialogue—a conversational agent (CA) that aims to support effective self-management of PTSD. In this work, we have focused on assessing the feasibility and acceptance of PTSDialogue for individuals living with PTSD.

**Methods:**

We conducted semi-structured interviews with individuals living with PTSD ( N=12 ). Participants were asked about their experiences with the PTSDialogue and their perceptions of its usefulness in managing PTSD. We then used bottom-up thematic analysis with a qualitative interpretivist approach to analyze the interview data.

**Results:**

All participants expressed that PTSDialogue could be beneficial for supporting PTSD treatment. We also uncovered key opportunities and challenges in using CAs to complement existing clinical practices and support longitudinal self-management of PTSD. We highlight important design features of CAs to provide effective support for this population, including the need for personalization, education, and privacy-sensitive interactions.

**Conclusion:**

We demonstrate the acceptability of CAs to support longitudinal self-management of PTSD. Based on these findings, we have outlined design recommendations for technologies aiming to reduce treatment and support gaps for individuals living with serious mental illnesses.

## Introduction

Post-traumatic stress disorder (PTSD) is a pervasive mental health concern. Millions of people suffer from PTSD, which is characterized by a range of distressing symptoms after exposure to traumatic events such as combat, sexual assault, natural disasters, or other life-threatening situations.^1,2^ According to the National Center for PTSD, approximately 13 million adults in the United States have PTSD in 2020, and 6% of the U.S. population will have PTSD at some point in their lives.^1^ PTSD symptoms usually occur within three months of the traumatic event, but can sometimes appear later.^3^ They can include re-experiencing the traumatic event, avoidance behaviors, negative changes in thinking and mood, and heightened arousal and reactivity.^3^ These symptoms can have a debilitating impact on mental health, physical health, work, and relationships of individuals living with PTSD.^4,5^ As such, it is imperative to support effective and timely management of PTSD symptoms.

Traditionally, the management and treatment of PTSD have relied on evidence-based treatments including one-on-one therapy sessions with mental health professionals.^6^ While these approaches have proven effective for many individuals, there remains a treatment gap resulting from different socioeconomic obstacles, such as structured barriers (e.g. financial) and attitudinal barriers (e.g. perceived stigma).^7,8^ Thornicroft et al.^8^ noted a high prevalence of structural and attitudinal barriers to obtain PTSD treatment. Specifically, the demand for specialized mental health services often exceeds the available resources, leading to long waiting periods for treatment.^9^ These barriers underscore the need for innovative and accessible approaches to supplement traditional intervention methods.^9^

Recent advancements in technology have explored new ways to bridge the treatment gap in PTSD care. eHealth technologies have been shown to improve access to care and reduce barriers to treatment.^10^ However, these technologies also face challenges such as low engagement rates^11^ and a lack of user-friendliness.^12,13^ Engagement in eHealth refers to the degree and quality of user interaction with technologies. Effective engagement is crucial for maximizing the benefits of eHealth interventions and improving health outcomes.^14^ To address these challenges, researchers have proposed using conversational agents (CAs) to support individuals living with PTSD.^15–17^ CAs have the potential to provide a range of benefits. First, CAs focus on high interactivity, which aims to facilitate a conversational approach rather than simply delivering instruction.^18^ Furthermore, CAs support social presence, such as empathy and anthropomorphism, which can contribute to enhanced user trust^19^ and engagement.^20^

Despite the potential benefits of CAs for supporting individuals living with PTSD, there is still a lack of understanding about using CAs to support continuous self-management of individuals with PTSD. Additionally, there is uncertainty about how to design CAs to support continuous self-management. To address this gap, we developed PTSDialogue—a CA to support the continuous self-management of individuals living with PTSD.^15^ We have designed PTSDialogue in collaboration with clinical experts to prioritize interactivity through customization of user preferences and conversational turn-taking to enhance the user experience, foster social presence, and improve sustained user engagement and adherence. PTSDialogue offers a wide range of support functionalities, including psychoeducational resources, assessment tools, and a diverse set of symptom management interventions.

In this paper, we assess the perceived feasibility and acceptance of PTSDialogue by conducting semi-structured interviews with individuals living with PTSD (N=12) to gain insights into their perceptions and experiences with using a CA designed to support PTSD self-management. All participants expressed that PTSDialogue could be beneficial for supporting PTSD treatment. Our findings establish opportunities and challenges in using CAs to complement existing clinical practices and support longitudinal self-management of PTSD. We also highlight important design features of CAs to provide effective support for this population, including the need for personalization, education, and privacy-sensitive interactions. Based on these findings, we have outlined design recommendations for technologies aiming to reduce treatment and support gaps for individuals living with serious mental illnesses.

## Related work

### PTSD treatment using eHealth technologies

In recent years, eHealth technologies have emerged for delivering interventions and support to individuals living with PTSD through Internet-based interventions (IBIs), and mobile applications (apps). IBIs for PTSD utilize computer programs to remotely deliver cognitive training, psychoeducation, interactive exercises such as creating trauma narratives, and resources. These interventions often follow a structured course format, prompting users to complete modules on a weekly basis.^21^ Kuester et al.^22^ evaluated the effectiveness of IBIs in improving PTSD symptoms and found moderate to strong effects, especially when compared with passive control conditions.

Furthermore, mobile apps designed for PTSD treatment offer a multifaceted approach to care, encompassing psychoeducation, symptom tracking, coping strategies, and crisis support. For instance, the Department of Veterans Affairs developed the mobile app, PTSD Coach.^23^ This app provides users with information on PTSD, offering guidance on symptom management and coping strategies. PTSDCoach can help to reduce the severity of symptoms for some individuals living with PTSD.^24^ This demonstrates the potential of mobile apps as self-management tools in mitigating the impact of PTSD on individuals’ daily lives.

While research has shown promising results in symptom reduction, eHealth technologies face challenges associated with sustaining user engagement and adherence.^25^ High user engagement and adherence are highly important for the success of interventions aimed at enhancing health outcomes.^26^ However, user engagement with eHealth technologies tends to decline over time, with only a fraction of users remaining active after several months.^27,28^ Addressing this issue and promoting sustained engagement is critical for maximizing the effectiveness of eHealth interventions in the context of PTSD.

### CA in mental healthcare and PTSD

CAs have shown promise in the field of mental health, including the treatment of PTSD. Recent studies have used CAs to provide support for a wide range of mental health issues including schizophrenia,^29^ depression,^16,30,31^ and anxiety disorder.^16,30,32^ CAs can be particularly useful in mental health assessment since they can lead to more effective self-disclosure.^33,32,16^ Recent work has explored CA attributes that can better support self-disclosure. Lee et al.^34^ reported that reciprocal disclosure from CAs can support deep self-disclosure from users. Cho et al.^35^ found that the use of backchanneling cues in CA interactions can lead to a perception of active listening, which was associated with more emotional disclosure from users.

Prior work has also used CAs to support coaching for self-management of mental health issues. For example, Morie et al.^36^ designed a CA to support veterans suffering from PTSD which aimed to provide a virtual environment for healing activities and social interactions. Tielman et al.^17^ designed a virtual coach to motivate and support individuals living with PTSD during therapy. Fitzpatrick et al.^30^ developed Woebot to provide psychotherapy and education focusing on depression and anxiety disorders. Other prior work has also explored the use of CAs to provide educational support. Swartout et al.^31^ created SimCoach to educate veterans and their families about PTSD and depression. It also provides tailored knowledge based on users’ needs, preferences, and concerns.

While there has been an increasing interest in using CAs for mental health support, there is a lack of understanding regarding how CAs can be designed for sustained user engagement and adherence.^18,37^ Specifically, limited work has focused on designing CAs to support longitudinal self-management of individuals with PTSD. Given the chronic nature of PTSD, understanding effective mechanisms for providing longitudinal support is critical to the success of technology-supported interventions. Our work aims to address this knowledge gap by evaluating the acceptability of PTSDialogue specifically for supporting the longitudinal self-management of individuals living with PTSD.

## Method

### PTSDialogue

**Figure 1.**
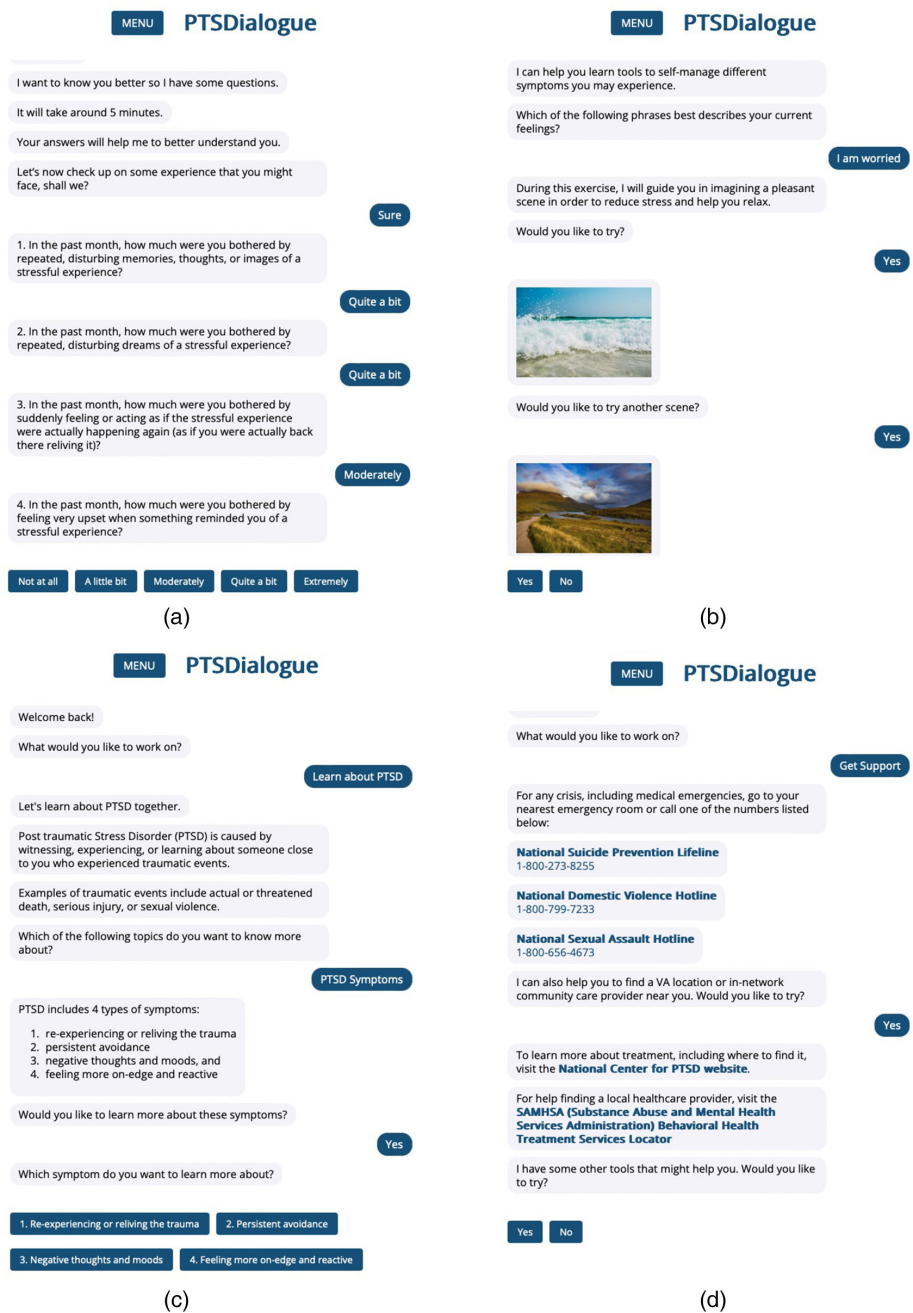
PTSDialogue is a self-management tool to support individuals living with PTSD based on conversational interaction. (a) The *Take assessment* module provides a checklist to assess symptom severity; (b) The content in the *Manage symptoms* module provides a number of strategies to manage post-traumatic stress disorder (PTSD) symptoms; (c) The *Learn about PTSD* module aims to inform users about causes, symptoms, and management strategies for PTSD; (d) The *Get support* module provides information for a number of resources (e.g., phone numbers to helplines).

PTSDialogue is a finite-state and web-based CA. Interactions in PTSDialogue are structured with a dialogue framework using content adapted from PTSD Coach.^27^ Design and implementation details have been described in prior work.^38^ It comprises of six distinct self-management modules (see Figure 1): “Take Assessment,” “Manage Symptoms,” “Get Support,” “Learn About PTSD,” “Track Progress,” and “Daily Symptom Checker.” The “Take Assessment” module offers a self-report assessment tool that measures symptom severity using the PTSD Checklist for DSM-5 (PCL-5).^39^ In the “Manage Symptoms” module, users can access a range of coping tools such as “Thought Shifting,” “Positive Images,” and “Ambient Sounds.” The “Get Support” module provides resources to find help near users. The “Learn About PTSD” module provides educational content, including the causes, symptoms, and effective management strategies for PTSD. The “Track Progress” module keeps a historical record of symptom severity, tracked through self-assessment scores. Lastly, the “Daily Symptom Checker” module conducts daily self-assessments to monitor distress levels effectively.

**Figure 2.**
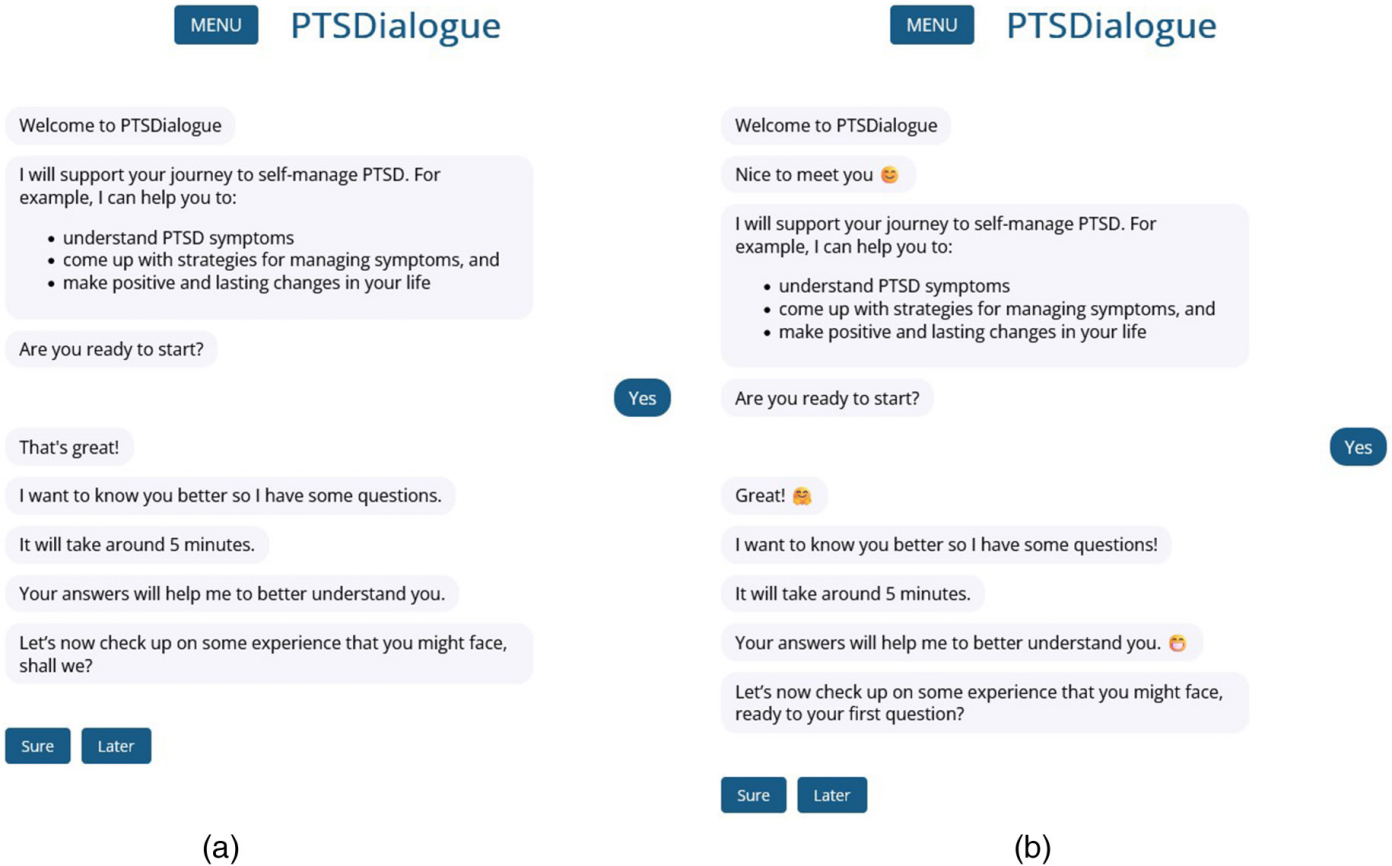
Interactions with both personas in PTSDialogue: the professional persona with a formal and neutral interaction style (*left*) and the friendly persona with an informal interaction style (*right*).

PTSDialogue has two distinct personas, each tailored to facilitate different interaction styles (see Figure 2). These personas aim to establish varied social presences—namely, a professional persona and a friendly persona. The professional persona emphasizes directness, precision, and formality while maintaining a neutral communication style. In contrast, the friendly persona aims to create a friendly and welcoming environment by adopting a casual conversation style that is enhanced by the usage of emojis. All messages align with the interaction style associated with the chosen persona, even as the message content itself remains consistent across both personas.

### Participants

To identify potential participants, we reached out to relevant communities by sending emails or sharing study flyers. We also used our connections with the National Center for PTSD and Hershey Medical Center to identify individuals living with PTSD. Potential participants were contacted through recruitment emails providing details about the study. We then conducted an additional screening test to confirm the eligibility of study participants. For screening, we used the Primary Care PTSD Screen for DSM-5 (PC-PTSD-5)^40^ and PCL-5.^39^ Both tests identify probable PTSD status based on self-reported scales. Participants were deemed eligible for the study if they self-reported experiencing PTSD symptoms of at least mild severity (minimum cut-off of 3 for PC-PTSD-5^40^ and 33 for PCL-5^41^). All participants are adults, aged 18 years or older, and English speakers. In total, we recruited 12 individuals living with PTSD. This sample size is consistent with prior work on evaluating the acceptability of CAs in the context of mental health.^42–44^ Table 1 shows the demographics of 12 participants.

**Table 1.** Demographics of 12 participants.

Participants	Gender	Age	Education level
P1	Male	50–75	Associate degree
P2	Female	35–49	Bachelor’s degree
P3	Female	18–24	Bachelor’s degree
P4	Female	18–24	Associate degree
P5	Male	25–34	Associate degree
P6	Female	25–34	High School
P7	Female	18–24	High School
P8	Female	25–34	Associate degree
P9	Non-binary	18–24	Bachelor’s degree
P10	Female	35–49	Associate degree
P11	Female	25–34	Associate degree
P12	Female	25–34	High School

### Procedure

Our primary objective was to assess the perceived feasibility and acceptability of utilizing PTSDialogue as a tool to facilitate the self-management of individuals living with PTSD. Specifically, we conducted one-on-one, semi-structured interviews with people living with PTSD, focusing on their perspectives regarding the usage of CAs in the context of PTSD support. Interview sessions were conducted remotely. Each interview lasted approximately 60 min. Participants received a $30 Amazon gift card as compensation.

During the interview, we first collected demographic information through a web-based survey. Then, we provided participants with access to the web-based PTSDialogue prototype, allowing them to interact with its various functionalities and features. We encouraged participants to articulate their thoughts and reflections as they interacted with the prototype, adhering to the “think aloud” methodology.^45^ Participants shared their screens so that the interviewer could see how they interacted with the prototype.

While participants engaged with the prototype, we posed follow-up questions to clarify their “think aloud” comments. We also employed a semi-structured interview script to systematically gather insights and feedback from participants.^46,47^ We specifically asked for their thoughts and feedback on the perceived feasibility and acceptability of PTSDialogue, as well as any suggestions for new features or recommended enhancements to improve its usability and acceptance. Our discussions expanded to look at how participants envision PTSDialogue being integrated into their existing clinical workflows and practices for PTSD management. The interviews concluded by eliciting participants’ feedback and recommendations directed toward technology designers and developers committed to assisting individuals with mental illnesses beyond PTSD. Data saturation was reached when we observed that no new insights were emerging from the data, with repeated patterns consistently identified across all participant responses.

We used bottom-up thematic analysis with a qualitative interpretivist approach to analyze all transcripts.^48^ Inter-rater agreement was achieved through two stages of analysis. In the initial phase, two coauthors independently went through each transcript and noted keywords within participant responses until no new keywords or themes emerged. The two coauthors then iteratively combined the identified keywords in the second stage and grouped them into themes. Follow-up conversations among these coauthors were used to resolve any disagreements regarding the themes that were generated.

### Ethics

The study was approved by the Penn State University institutional review board (STUDY00021763). We obtained written informed consent from participants before collecting any data. To protect participant privacy and confidentiality, the study data were deidentified. We used randomly generated numbers as identifiers for subsequent data analysis. To further enhance privacy, we did not collect any video data during the interviews. Access to the gathered study data was restricted to authorized members of the research team.

## Findings

Following the qualitative data analysis, we have identified four themes: acceptance of PTSDialogue to support PTSD care, the feasibility of integration with existing clinical practices, the potential for reducing health inequity and overcoming access barriers, and identified needs for CAs to support longitudinal management of PTSD. In the following sections, we discuss these themes and relevant subthemes.

### Acceptance of PTSDialogue to support PTSD care

All 12 participants agreed that PTSDialogue could be beneficial for supporting PTSD treatment. P2 stated that

I believe that [PTSDialogue] will be very beneficial for people who have experienced PTSD. […] The way that the typing and the information came out was very good, and it was very informational and educational, and everything was in good order.

P4 noted that PTSDialogue might be particularly useful for people who are new to PTSD and are experiencing situations for the first time because they could use it on a daily basis: “Especially for people first being introduced to PTSD and first initially experiencing events, it’s really helpful, and I think, like the daily check-in, and like progress tracking thing is pretty neat and helpful to do.”

Participants appreciated the potential for PTSDialogue to enhance accessibility and convenience by removing geographical and logistical barriers. Furthermore, by providing immediate access to resources, PTSDialogue can effectively support individuals with PTSD who may experience symptoms at any time. P8 highlighted the significance, stating, “I love the idea of it [PTSDialogue] because we don’t always have access [to treatment].” P4 also liked PTSDialogue because “It’s like talking to a virtual mental health provider. And then I like how this one had a lot of different options for you, and you could use it as a daily use.”

#### Conversational branching

PTSDialogue supports predefined options with distinct branches. In the context of CAs, branching is used to guide a conversation along different paths based on the user’s responses. Four participants underscored the value of branching for enhancing engagement by promoting ease of use. P12 highlighted that branching with predefined options “wasn’t difficult. It wasn’t difficult to read. It wasn’t difficult to understand. It was very, very easy.” P5 also emphasized that branching with predefined options is

Extremely beneficial, especially when it’s right at your fingertips. Some websites make you click a bunch of things and they just send you to different sites. That’s stressful. But [PTSDialogue] is extremely useful. It is user-friendly where it’s very clean and concise.

Similarly, P9 expressed the ease of making rapid decisions, particularly when faced with options laid out for selection: “I can quickly decide what I want when I have clear options presented to me. It’s convenient because it requires minimal effort and thinking, which is especially helpful when I’m feeling distressed.” P4 echoed this sentiment, noting that predefined branching simplifies engagement by reducing the cognitive load associated with decision-making:

This one didn’t show you that [show all choices when you first open it]. That makes it kind of easier for engagement, because, you have to make that little choice to click on the button to see your actual progress, so I feel like it’s not really a deterrent. You still have to stick to it.

#### Persona

Participants also appreciated the integration of different personas within PTSDialogue. Each persona offers a diverse range of conversational styles and tones, enhancing user engagement. For example, one persona adopts a straightforward and precise demeanor, employing a formal and neutral communication style, while another persona shows cheerfulness and friendliness, engaging in informal interactions and emojis. P1 noted that this diversity allows users to choose the persona that resonates best with their individual preferences, promoting engagement: “I think they’re both needed. Because our ways of learning are different. Our learning styles are different. So, having two personas is going to be comparable and helpful to many people across all different types of spectrums, education levels.”

The majority of participants expressed a preference for the cheerful and friendly persona, noting that it was “a lot more engaging” [P5] and made interactions “feel slightly more personable” [P7]. In particular, the use of emojis emerged as a significant factor in establishing a connection between users and PTSDialogue. P3 mentioned that “emojis was nice because it gave some life.” As P4 observed, “the friendlier one [persona] uses emojis appropriately, and everything is very encouraging. […] So, the friendlier one would be nice for people, especially just for starting out.”

However, it is essential to strike a balance when developing personas. P3 highlighted the importance of ensuring that expressions and interactions remain applicable and contextually relevant. P3 shared experiences with other mental health chatbots that attempted to be excessively friendly, sometimes to the point of being overwhelming or feeling too personal, which could be disconcerting for users. In contrast, PTSDialogue aimed to strike a balance by maintaining friendlier language without becoming overly personal or intrusive, making it more appealing and comfortable for users seeking support:

It’s interesting because I’ve used mental health chatbot before and I feel like they tried to make it almost too friendly to the point […] It would send like gifts, or it like something like funny, mean, but other times it felt weird because it is a chatbot, and it’s too personal and friendly, it is kind of an AI. But I liked this one [PTSDialogue]. It [has a] friendlier language, but it wasn’t like anything out of pocket. emojis were nice. […] It wasn’t, too, over the top personal […] I said the other [chatbot] was sometimes just overwhelming, or it was just too much sometimes. [P3]

#### Education materials

PTSDialogue aims to inform users about causes, symptoms, and management strategies for PTSD. Educational materials can enhance acceptance of PTSDialogue by making individuals feel more informed. P3 commented:

You like to learn more about PTSD. Although coping skills are important, sometimes they’re really hard for me to actually implement. So I do like learning more about PTSD facts and how [it] can relate to other mental illnesses and stuff like that.

P5 echoed this sentiment, expressing appreciation for the inclusion of factual information because “[My providers] don’t really explain much other than hearing symptoms, and explaining that you know your trauma caused it. So having that, it’s actually really nice.”

Furthermore, educational materials increase confidence, providing a sense of empowerment for people seeking help. P4 highlighted the utility of resources, especially for people navigating traumatic events for the first time, stating, “the learning about PTSD is really useful because you can get a lot more connected for specific help tailored to you.” P5 reinforced that educational resources

Give you an idea. Hey? You’re not alone, you’re learning about what’s going on. Not just. You have these symptoms, and you have this, you get to actually read more about it, especially with the fact that you’re not the only one that has it.

### Feasibility of integration with existing clinical practices

#### Providing just-in-time support for longitudinal management

PTSDialogue can provide an opportunity to bridge the gap between the immediate need for PTSD care and the limited availability of treatment resources, which are caused by geographical and logistical barriers. This is particularly critical for individuals who cannot find immediate support. Three participants emphasized how PTSDialogue may reach people in need regardless of time or place by delivering crucial resources such as coping tools and psychoeducation. P7 articulated the significance of continued availability of resources: “it’s a relatively good idea, especially in times of crisis where someone can’t immediately reach their therapist or someone else to talk about, but where it’s not an emergency where they would have to call 911.” Similarly, P10 shared experience and highlighted the advantage of being able to use PTSDialogue immediately:

When it was really bad, I called or texted the suicide number found online because I didn’t know the person. For all I know, I needed that conversation. And that was very helpful for me. I was on there for probably an hour, talking to somebody, and I mean, it was very basic. Similarly, [PTSDialogue] was probably scripted, but it was nice.

Furthermore, P4 emphasized the utility of PTSDialogue, particularly for individuals who are not in frequent therapy sessions, highlighting its potential to address the needs of patients seen on a less frequent basis, perhaps once a month or every two weeks:

[PTSDialogue] could be useful, especially for patients who aren’t, super, frequently seen in therapy, or, they are seen every once a month or 2 weeks. But they or their provider still wants them to kind of have those check-ins. [PTSDialogue] will be a really useful tool for them. Just so that they can track that, and when they feel like they need something not necessarily like a whole appointment, they can use this to check in with themselves.

#### Addressing stigma and treatment-seeking hesitancy

Participants noted how stigma and treatment-seeking hesitancy affect their perceptions of formal treatment with clinicians. They highlighted how PTSDialogue can alleviate these concerns by serving as a facilitator of the transition to traditional PTSD care, which could be particularly beneficial for individuals who initially hesitate to seek formal treatment. For example, P10 highlighted the potential benefits of PTSDialogue, particularly for those who may be unwilling or unable to engage in one-on-one conversations or face-to-face interactions during moments of emotional distress such as when experiencing anger related to PTSD:

[PTSDialogue] could be helpful, especially for people who aren’t ready to talk one-on-one with somebody, or just maybe that moment they’re not ready to sit face-to-face someone or they can’t. I know with some of my things like my PTSD, especially whenever my anger takes a while, but I can’t break it. I know that sometimes I don’t want to talk to a person. I don’t want to admit that I’m broken. So, I like [PTSDialogue].

P2 further emphasized that sharing sensitive information, such as the details of a traumatic experience, may be more acceptable with PTSDialogue, as opposed to divulging such information directly to a clinician:

I think the information regarding whatever circumstance that you had. If you experience a sexual assault or some kind of negative symptoms, I think it would be okay to share the symptoms and your experience, and what happened on the night of the sexual assault. I think that would be okay to share with the chatbot more than with a clinician because the clinician can get in the way.

PTSDialogue can potentially be useful for individuals who are not yet prepared to engage with in-person PTSD treatment: “[PTSDialogue] would be awesome because I know there are some days where I don’t even want to go into therapy because I just don’t want to talk” [P10]. Importantly, engaging with PTSDialogue can foster a sense of comfort and readiness to transition toward in-person treatment: “if they’re hesitant to seek treatment, you could share this with them as a way to get comfortable with mental health treatment. I feel like that could be really helpful, too” [P4].

Moreover, the ability of PTSDialogue to track users’ progress is especially beneficial for individuals who may encounter challenges in effectively communicating with their healthcare providers. P5 underscored this by emphasizing that PTSDialogue can serve as an auxiliary tool for those who struggle to articulate their experiences with their care providers: “It’d be a good way for people who don’t really communicate well with their provider. It’d be a good way for them to keep track when they’re not in their care to see how they’re doing” [P5].

PTSDialogue can offer an anonymous and nonjudgmental environment for individuals living with PTSD affording them the freedom to discuss their thoughts, emotions, and experiences without the fear of external judgment. P8 emphasized the significance of PTSDialogue as a method of explaining PTSD symptoms without fear of being assessed by others, adding that “it’s a good idea to help explain the symptoms without having to be judged by somebody else.” P10 further highlighted the necessity of PTSDialogue for those who find it challenging to engage in direct communication, either by phone or face-to-face. Despite the noticeably scripted nature of the interaction, P10 appreciated the liberating aspect of not having to confront someone in person and potentially feel judged:

I wasn’t able to call and speak to somebody. I wasn’t able to talk to somebody face to be. You could tell it was probably scripted, but it was nice because I didn’t have to be face-to-face with anybody. I could just be honest and raw and not feel like I was being judged. So if people are having problems with that, I think that the computer system might be a good stepping stone. It’s not going to solve everything.

Similarly, P2 said they would be more likely to communicate their symptoms and experiences with PTSDialogue than clinicians because it eliminated the perceived potential for the clinician to interfere or pass judgment on their experiences:

I think it would be helpful to use [PTSDialogue]. I think it would help better than the clinician would help. It makes a very quiet atmosphere. [PTSDialogue] doesn’t judge you and make you feel like you did something wrong and doesn’t provide any feedback.

### Potential for reducing health inequity and overcoming access barriers

Marginalized communities encounter substantial obstacles in accessing mental healthcare. This is true for individuals with PTSD as well. For example, P1 noted “the historical mistrust of the healthcare system” among African Americans, irrespective of whether the interaction is with a chatbot or a human:

Trust in the healthcare system has been very traumatizing historically. Whether it’s interacting with a chatbot or a real person, our focus remains on the fact that this is part of the medical community. We want assurance that someone within the medical community is handling the information and data behind the scenes, and this association is crucial to us.

P1 emphasized the need for mitigating biases against racial minorities, including African American individuals: “Developing technologies that are not biased towards African American people and other racial minorities in the country make feel us awesome.”

P1 also identified challenges associated with gender especially, masculinity within the male community, which can result in a reluctance to discuss mental health concerns:

To get us to talk about mental health is something that very rarely we would even talk about, because it’s not seen as something that’s manly in our community [male community], and actually it is challenging for men in general, because men somehow are wired to think that if we need any type of help, especially mental health help, then, we kind of look at that, as like. I’m strong enough to get through this myself. I don’t need anyone to talk to.

P1 advocated for “breaking through this barrier,” recognizing the potential of PTSDialogue to provide valuable support to men who may initially believe they can handle PTSD symptoms just by themselves.

Marginalized communities face a multitude of barriers when it comes to supporting mental health, and age can be a particularly notable obstacle. P4 emphasized that PTSDialogue has the potential to play a pivotal role in reducing health inequity among young people dealing with PTSD: “[PTSDialogue] would be useful, especially with younger people. It could be really helpful to promote it on campuses, especially related to sexual abuse. And then recommend using [PTSDialogue] and counseling and treatment centers.”

PTSDialogue can help us to alleviate obstacles to PTSD care within marginalized communities. By providing accessible resources, it addresses the specific needs of individuals who may lack access to traditional PTSD care. P1 highlights the significance of these resources, particularly in supporting underserved populations: “Having resources available to underserved populations in any shape or form is going to really help.” Also, P1 recommended focusing on mitigating any biases during the development of PTSDialogue because “people can feel comfortable and secure, knowing that their feelings are being taken seriously and not overlooked.”

### Identified needs for CAs to support longitudinal management of PTSD

#### Personalization

Personalization is recognized as a fundamental element in the effective management of PTSD, enabling tailored treatment that addresses individual needs and experiences.^49^ It leads to enhanced engagement, treatment efficacy, and reduced barriers to seeking help. Four participants underscored the significance of inquiring about trauma type, recognizing its potential in improving individual experiences. However, they also noted the importance of balancing specificity, which involves asking detailed questions, and sensitivity, which involves asking questions in a respectful and nonintrusive manner. P9 suggested framing questions with sensitivity, allowing respondents to share without feeling intrusively probed, for example, “Do you suffer from PTSD because you have like prolonged exposure to trauma?” P12 introduced the idea of advanced needs assessment, acknowledging the varying degrees of PTSD severity: “It would be helpful that there’s mild, moderate, and severe when it comes to someone’s illness and someone’s PTSD.” P10 advocated for more specific inquiries, emphasizing the openness of individuals to share their experiences:

More specific questions would help with better answers and assistance. Most people are going to be open to answering honestly. I don’t think that you’d have to worry about being too intrusive because we’re here to help. I know, a lot of people are worried about tiptoeing around most of the time. It’s not us. We just want someone to try to understand us. And more questions would be a way to try and get to understand.

Understanding diverse backgrounds and needs is crucial, as emphasized by P6, who suggested broadening the inquiry to encompass various trauma types such as military service and experiences such as child abuse. Additionally, P6 highlighted the importance of considering factors such as health insurance coverage and location diversity:

During the assessment you could ask, are you military, or is it like child abuse PTSD, or whatever? And then you could ask, do you know what type of health insurance you have? And then [PTSDialogue] could say I can show your medical assistance covers for in your state, because that’s the other thing in a lot of states have different laws and stuff.

P8 also proposed location-based personalization while asking for a “ZIP code that gives people an area, and might be a little bit more personable resource.”

Three participants highlighted the need for personalized measurement tools for symptom management. P4 suggested that personalized measurement tools could be a valuable resource for offering symptom-managing activities tailored to individual needs, rather than simply presenting repetitive tools: “The symptom management tool showed the same ones most of the time. I think [personalizing] would be really helpful with simple measurement tools.” P7 recommended incorporating tools that enable users to save information upon completing activities, thus establishing a repository of personalized coping strategies. Such functionality could also facilitate learning about users’ needs over time:

It’d be interesting to see a way to save some sort of information. So once you’ve done one of the activities that help manage your symptoms, you can save the information that it tells you so that you can use it alongside [PTSDialogue] without always having to sort of learn these things over time.

Similarly, P9 envisioned a system where users express their preferences, allowing the CA to queue up relevant information based on individual interests: “If [PTSDialogue] asked you what you’re most interested in so that it would have it queued up for you later, that’d be cool.”

#### Self-tracking

In the context of managing PTSD, our participants emphasized the significance of tracking progress as a personalized tool for gaining insights into symptom management strategies. This process entails not only assessing personal scores and mood but also monitoring the utilization of symptom management tools.

Regularly tracking one’s progress allows individuals to reflect on their PTSD symptoms and their responses to various coping techniques. It provides feedback on their treatment showing whether a particular treatment approach is suitable for an individual’s needs. As articulated by P6,

The track progress would be really good because if I go to therapy and then I go through these questions once every 3 months, for example, I can see if therapy is helping or do I need to look into a different treatment to get improvement.

Moreover, progress tracking provides users with opportunities to identify effective strategies. By monitoring their experiences and symptom management efforts, users can discern which strategies are most effective for them. This not only enhances their overall engagement with the treatment process but also encourages individuals to explore symptom management tools they may not have previously considered. By analyzing patterns within their progress data, users may identify areas where alternative strategies could be beneficial. For example, P5 noted the benefit of having all relevant information in one place, which could inspire them to experiment with new approaches to symptom management:

It tracks what you’re doing and you’re like, Well, I did this. Let’s say this was the different grounding technique I use. I think it’s useful. I said that those grounding things are absolutely for me a critical thing because I do the whole like sounds escape stuff. But I’m trying to get out of an episode and the pictures. I never thought about doing that. Let’s say something I personally will start doing and just trying to imagine stuff. But having that all [in] one space would be great for someone that’s still going through, you know, treatment and hasn’t had an option to the opportunity to do try that.

However, it is important to acknowledge that tracking progress may pose challenges depending on the user’s current status. As highlighted by P3, individuals on a positive trajectory who begin to plateau or experience declines in their progress may find this discouraging:

I have mixed emotions about it. On one hand, it’s good to track progress and all, especially on rough days. If the app prompts you to journal or do something like that, it could really help. But on the other hand, if you’re doing well and then suddenly hit a plateau or start to decline, seeing that could be discouraging. So, it’s a bit of a mixed emotion. Overall, though, I think it could be helpful.

Therefore, while progress tracking offers numerous advantages, it should be implemented with consideration for individual progress.

#### Sharing data with healthcare providers

Participants in our study highlighted the potential benefits of integrating data sharing with healthcare providers from a user-centric perspective. They recognized that integration could support more equitable, patient-centered care while enabling improved workflows. Participants acknowledged that there are times when they may withhold important information from their clinical support team due to reticence or stubbornness. In such cases, sharing data can bridge this communication gap, offering clinicians valuable insights into patients’ health and activities:

I think [data sharing would] be really good, because sometimes I don’t voice certain things to my providers and letting them see what my activity is. It might help them take care [of] me because I’m pretty stubborn when it comes to my care. Sometimes I don’t talk about stuff. So they can see that they can pick up on that. I think that’s a great thing. [P5]

However, it is important to note that not everyone may share this perspective. Some individuals, particularly those who prefer not to be judged by a person, may not want data sharing for the purpose of integrating with existing clinical practices in PTSD care: “I don’t think it’s okay because PTSD is too hard to deal with for clinicians. They could judge you or tell you the wrong answer.” [P2]

#### Navigating privacy and ethical concerns

Addressing privacy and ethical concerns is critical to ensure the successful adoption of health technologies. This is especially important for individuals with serious mental illnesses, including PTSD, due to the risk of stigma. Five participants expressed a positive view on sharing data with existing clinical practices. P3 underscored the potential benefits of sharing data with healthcare professionals. P3 viewed data sharing as a means to provide valuable information to professionals, enhancing the overall quality of care: “I feel like anything would share. It’s gonna benefit whatever professional that you’re working with. […] All the information that I saw would make sense for someone to share with the doctor.” P4 further elaborated on the potential benefits, noting the alignment between the questions posed by PTSDialogue and those typically encountered in clinical settings:

It would be pretty helpful because it’s basically the same questionnaires that your actual provider would give you. […] Those are very standard questions, so I feel like it translates very well into the tracking. If this would be something that someone wants to share with their actual provider, it could be really helpful.

Furthermore, P7 emphasized the significance of data sharing, seeing it as a tool for clinicians to gain insights into a user’s emotional well-being and enabling more effective interventions:

It’s a good idea for it to be a tool that is shared with clinicians and therapists, and such just because I think this would be a good way for them to see how you feel and maybe touch base on it.

Three participants emphasized the need for a robust consent process. P6 stressed the importance of transparency, drawing parallels with traditional mental health treatments where consent forms are standard practice:

When it comes to what information a [PTSDialogue] will collect, it’s essential to establish consent. In any other traditional mental health treatment I’ve experienced, I’ve had to sign a consent form either on paper or on an iPad. It’s similar to visiting a doctor where we share information, and this is covered by HIPAA (Health Insurance Portability and Accountability Act) for consent. So, clarity is upfront information for users.

The consent process should align with established guidelines, such as those outlined in HIPAA, and should provide users with a clear understanding of what data would be shared, how it would be processed, and ensure that the process adhered to privacy regulations. P1 also recognized the value of the consent process, emphasizing its role in enhancing patient care:

You will likely request consent before diving into that particular area, so you’ll branch off and ask for consent. This involves adhering to HIPAA guidelines and obtaining the individual’s permission. You will thoroughly explain the process to them and proceed from there. When medical professionals receive this information, I believe it will be a valuable asset in treating the patients.

P7 echoed the need for privacy, emphasizing the importance of data encryption and compliance with HIPAA regulations: “I personally feel uncomfortable [if] my data is not private. So obviously, I assume when it’s being shared with people like to a therapist, and such it would fall under like HIPAA. They keep the data encrypted.”

In contrast, P2 expressed concerns regarding data sharing, particularly for individuals with specific trauma experiences:

it might not be right to have the person share their experience, because unless it was witnessing a death or some kind of veteran experience. But when it’s a sexual assault it might not be ethical to share that information.

To solve privacy or ethical concerns, building trust is a critical component of effective PTSD care because it can help patients feel more comfortable sharing their experiences and feelings. As P1 points out, while CAs “are going to be used in an increasingly high manner in the future,” building trust within communities is important. Trust is a fundamental concern in the healthcare industry, and nurturing it is essential for ensuring patients can fully benefit from emerging technologies while feeling confident in their safety and care.

#### Supporting multimedia interactions

Participants noted the need for better integration of visual and audio resources in PTSDialogue. Visual and audio elements play a pivotal role in enhancing user engagement and tailoring support to diverse learning styles and preferences. Participants have expressed their enthusiasm for visual resources such as images and videos. P1, for instance, emphasized the importance of these visual elements, noting that they cater to different learning styles worldwide: “Being able to see the beach,” P1 elaborated, “is something that works across different learning styles of people all around the world.” Visual learners find value in having these images as they provide a tangible point of reference. P2 even suggested going a step further by providing descriptions beneath pictures, making the visual content more informative and accessible, for example, “if it’s a picture of a serene scene, like waves gently rolling in the sunlight, having a video showcasing that could be helpful.” Furthermore, the use of audio resources has the potential to increase accessibility and engagement. P7 emphasized the importance of instructional audio, suggesting that CAs could guide users in immersive sensory experiences by instructing them to “close your eyes and imagine hearing the sound of the ocean and smelling the saltwater.” P1 commended the idea of combining visual and audio elements, stating that

Maybe even for people who might not be visual learners, maybe even having the ability to play audio to read back information from the graph and explain it to us so we can view it with visual or with audio plus.

Participants also expressed a desire for more in-depth engagement with visual and audio resources. P9, for instance, proposed enriching activities by providing specific instructions for users to imagine sensory details related to the visual scenes presented. This immersive approach can deepen the impact of such activities, making them more therapeutic and effective. P7 suggested that activities include detailed instructions, thereby guiding users through a multi-sensory experience that aligns with therapeutic practices they may have encountered in traditional therapy.

#### Complementing existing clinical practices

CAs offer valuable support in the context of PTSD care, extending their reach to provide ongoing assistance and education. However, it is important to recognize that CAs should complement, not replace, clinicians to provide PTSD care. Some participants echoed the view that CAs cannot replace professional therapy. P3 emphasized the importance of having disclaimers that clarify that PTSDialogue “does not replace professional therapy.” While users can get responses instantly, having a personal therapist provides a vital outlet.

Moreover, there was an acknowledgment that CAs may encounter technical issues or situations that demand human intervention. P6 highlighted the need for real human support when issues go beyond predefined responses or when technical glitches occur:

It would be helpful to have the option to connect with a real person during their office hours. For example, if I encounter an issue with [PTSDialogue] or if there’s a technical problem, having the option to reach out to a human for assistance would be beneficial.

This human-in-the-loop approach ensures that individuals can seamlessly transition to human support when necessary, maintaining the safety and effectiveness of the care system.

Another crucial aspect to consider is the potential exclusion of individuals who are inexperienced with technology or lack access to technology. While CAs can offer valuable support, they rely on digital interfaces that may pose a barrier to those who are not comfortable with computers or smartphones. P6 pointed out that inexperience with digital technology is a concern for some individuals, emphasizing the importance of offering support for technical issues and mental health assistance. P1 also raised concerns about serving individuals without access to digital devices, highlighting the need to address this challenge in order to provide equitable care: “I find it helpful. However, the challenge might be for those who don’t have access to computers, iPads, smartphones, and such devices. How do we support those individuals who lack access to such technology?”

## Discussion

In this study, we assessed the acceptance of using PTSDialogue to support individuals living with PTSD. During the study, we conducted interviews with 12 people living with PTSD. All participants agreed that PTSDialogue can be used to support self-management of PTSD care. We also identified key features and interactions supported by PTSDialogue that contribute to supporting self-management for our target population. Based on these findings, we discussed how acceptance of CAs can be improved in longitudinal mental healthcare and provided design recommendations for future CAs aiming to support self-management of individuals with mental illnesses beyond PTSD.

### Navigating trust and human collaboration in CAs for longitudinal support

#### Trust toward CAs

Privacy and ethical concerns play a significant role in integrating CAs into longitudinal mental healthcare, particularly for individuals with PTSD. In our study, we gathered valuable insights into privacy concerns related to data sharing for CAs supporting the longitudinal management of PTSD. Participants expressed positive views on sharing data with existing clinical practices. They emphasized the potential benefits of sharing data with healthcare professionals, such as enhancing the overall quality of care and enabling more effective interventions. However, participants also highlighted the need for a robust consent process and transparency regarding data collection and sharing. Others raised critical ethical considerations, suggesting that data sharing might not be suitable for individuals with specific trauma experiences.

Addressing these privacy and ethical concerns requires a focus on building trust, which is essential for effective PTSD care. Trust plays a crucial role in enabling people living with PTSD to feel comfortable sharing their experiences and emotions, ultimately enhancing their overall care experience. This involves implementing robust data security measures, ensuring compliance with privacy regulations such as HIPAA, and transparently communicating how user data will be collected, used, and protected. By fostering trust, users can feel more confident in handling their personal and sensitive information, thus facilitating the integration of CAs into longitudinal mental healthcare practices.

It is also important to recognize the diversity of experiences and trauma backgrounds among individuals with PTSD individuals with PTSD, as well as those experiencing PTSD symptoms who do not meet the clinical threshold for a formal diagnosis. Some participants in our study expressed concerns that data sharing may not be suitable for those with specific trauma experiences, highlighting the need for personalized approaches and sensitivity in addressing privacy and ethical considerations. Future work should investigate how the efficacy of CAs might differ across different subpopulations in PTSD. Overall, fostering trust is crucial as CAs continue to play a role in supporting individuals with PTSD and other mental health conditions over time.

Furthermore, addressing privacy and ethical concerns is critical to ensure the successful adoption of health technologies aimed at supporting marginalized communities. Marginalized communities encounter substantial obstacles in accessing mental healthcare, with racial and ethnic minority groups facing heightened challenges, as outlined by the Centers for Disease Control and Prevention.^50^ Barriers include the difficulty in finding culturally competent providers, financial constraints, and the pervasive stigma surrounding mental healthcare. Additionally, racial discrimination and violence contribute to stress and racial trauma, further impacting the mental health and emotional well-being of individuals within marginalized communities.^50^

To effectively support marginalized communities in providing longitudinal mental healthcare, it is essential to prioritize building trust due to the heightened risk of stigma. CAs should be designed with empathy and a nonjudgmental approach to foster trust and improve access to mental health resources for marginalized communities. By addressing privacy and ethical concerns while fostering trust, CAs can play a crucial role in bridging the gap in mental healthcare for marginalized populations.

#### Human-in-the-loop

It is important to recognize the role of CAs as complementary tools rather than substitutes for human clinicians in managing PTSD. Participants emphasized that CAs should not intend to replace professional therapy. This finding aligns with previous research showing that patients are more likely to engage and share when they perceive technology as a reliable and supportive entity in their care.^51^ This understanding can promote acceptance of CAs as valuable adjuncts to professional therapy. Future work should focus on establishing a robust evidence base regarding how the efficacy of CAs compare with other eHealth technology platforms, when it comes to supporting self-management in PTSD. For example, there is an opportunity to conduct a future study using PTSDialogue and PTSD Coach to compare their relative efficacy in sustaining user engagement and optimizing clinical outcomes.

It is imperative to underscore the necessity of a human-in-the-loop approach, not only with clinicians but also with individuals who can support those who are inexperienced with technology or lack access to technology. This approach ensures that individuals can seamlessly transition to human support when necessary, maintaining the safety and effectiveness of the care system. For example, P6 highlighted the need for real human support when issues go beyond predefined responses or when technical glitches occur. Our participants suggested having the option to connect with a human for assistance. Additionally, it is critical to address the potential exclusion of individuals who are inexperienced with technology or lack access to technology. While CAs can offer valuable support, they rely on digital interfaces that may pose a barrier to those who are not comfortable with computers or smartphones. Therefore, offering support for technical issues and mental health assistance is essential. P1 also raised concerns about serving individuals without access to digital devices, highlighting the need to address this challenge in order to provide equitable care.

Therefore, the development of CAs should involve collaboration with humans, including mental health professionals, to ensure that the technology aligns with established therapeutic practices and ethical guidelines. This collaborative approach can enhance the acceptance and effectiveness of CAs in PTSD care by providing seamless support and addressing the diverse needs of individuals.

### Design implications

We provided design recommendations for future CAs aiming to support the self-management of individuals with mental illnesses beyond PTSD. First, personalized conversational dynamics are essential for tailoring interventions to individual needs and sustaining user engagement. Second, transparency in data sharing is crucial to address privacy concerns and foster trust among users. Third, providing multimedia messages, including immersive educational materials, is vital for enhancing user engagement and providing comprehensive support for the self-management of individuals with mental illnesses beyond PTSD.

#### Personalized conversational dynamics

To sustain user engagement, personalization is a critical element in the success of CAs designed to support the longitudinal management of PTSD care. It plays a pivotal role in tailoring interventions, leading to improved engagement and reduced barriers to seeking help. Participants expressed enthusiasm for the personalization capabilities of the CA, providing concrete examples of how tailored resources could significantly improve their experience. They stressed the importance of providing tailored coping tools that address individual needs and preferences. Additionally, participants highlighted the value of location-specific resources, such as local support groups or therapy options, that align with users’ unique experiences and geographic locations. Acknowledging the variations in state support and insurance coverage can impact user engagement.

Moreover, participants highlighted the value of CAs learning user preferences and adapting resource suggestions accordingly. They emphasized the significance of CAs recognizing individual preferences over time, allowing for a more personalized and effective support experience. For instance, participants suggested that CAs could learn from users’ interactions and feedback, such as which coping strategies they find most helpful or which types of resources they prefer. By continuously analyzing user interactions and preferences, CAs can refine their recommendations to better align with users’ unique needs and preferences.

Implementing this feature involves utilizing user input and incorporating user interactions and preferences. CAs can gather information directly from users through surveys, preference settings, and ongoing interactions. For example, users could indicate their preferred coping techniques, topics of interest, or specific resources they find helpful. The CA then stores this information and uses it to tailor future interactions and resource suggestions. By regularly updating and refining these stored preferences based on user feedback, the CA can continuously improve its ability to provide personalized support. Thus, we recommend designers allow CAs to learn from user interactions to enhance personalization.

Also, participants underscored the significance of progress tracking as a personalized tool to gain insights into symptom management strategies. Regular progress tracking enables individuals to reflect on their PTSD symptoms and their responses to various coping techniques. It provides valuable feedback on treatment efficacy, helping users assess whether a particular approach aligns with their needs. Progress tracking empowers users to identify effective strategies. By monitoring their experiences and symptom management efforts, individuals can pinpoint which strategies work best for them. This not only enhances their engagement with treatment but also encourages the exploration of new symptom management tools.

However, achieving this balance is crucial. Incorporating features that provide contextual feedback and encouragement can help mitigate potential feelings of discouragement. By framing progress tracking as a tool for empowerment and self-awareness rather than solely for monitoring outcomes, users may feel more motivated and supported. Moreover, integrating mechanisms for periodic review and reflection can help users contextualize their progress within the broader context, fostering a sense of continuity and purpose. Ultimately, achieving balance requires a nuanced approach that considers both the benefits and potential drawbacks of progress tracking.

Furthermore, integrating personalized personas within PTSDialogue offers substantial benefits. These personas employ diverse conversational styles and tones, enhancing user engagement and trust. All participants favored cheerful and friendly personas, emphasizing that they made interactions more engaging and personable. The use of emojis, in particular, played a significant role in establishing connections between users and PTSDialogue. However, a balance in persona development is essential. P3 highlighted the importance of ensuring that expressions and interactions remain contextually relevant. Participants appreciated that PTSDialogue maintained a friendly tone without becoming overly personal or intrusive, making it more appealing and comfortable for users seeking support. Thus, we recommend designers find a balance when developing personas’ characteristics.

#### Considering transparency of data sharing

Addressing privacy concerns in data sharing for CAs supporting longitudinal PTSD management, our study has provided useful insights. Participants’ perspectives provide a comprehensive understanding of privacy and ethical considerations. P4 highlighted the ubiquity of data sharing, emphasizing the importance of user comfort when sharing sensitive mental health information. The acceptability of data sharing hinges on users feeling at ease with the process. For considering the transparency of data sharing, participants emphasized the need for a robust consent process.

Therefore, we recommend that designers consider several key aspects to ensure the integrity and transparency of data handling within CAs. Firstly, data encryption and compliance with regulations such as HIPAA are essential components to protect user privacy and maintain data security. By implementing robust encryption protocols and adhering to regulatory requirements, designers can minimize the risk of unauthorized access to sensitive user information.

In addition to encryption and regulatory compliance, ensuring meaningful consent in CA design is paramount. Designers should strive to provide users with clear and comprehensive information regarding data collection, storage, and usage practices. This includes transparently communicating how user data will be utilized and shared, as well as offering users the opportunity to provide informed consent before their data is accessed or utilized. Moreover, designers should facilitate ongoing consent management mechanisms that allow users to modify their preferences and permissions over time, reflecting changes in their preferences or comfort levels. This can be achieved through regular prompts or reminders for users to review and update their data-sharing preferences that enable users to easily modify their settings as needed. By empowering users to maintain control over their data-sharing preferences and ensuring transparency in the data-handling process, designers can foster trust and confidence among users, thereby promoting successful adoption and long-term engagement with CAs in PTSD care.

#### Supporting multimedia interaction

Our research highlights the crucial need for immersive educational materials in the effort to provide comprehensive support for the longitudinal management of PTSD. These materials have the ability to effectively engage people while also educating them. Participants emphasized the need to learn more about PTSD beyond coping tools. Furthermore, our findings highlight that educational materials significantly enhance users’ confidence in seeking help. These resources provide individuals with a deeper understanding of PTSD, its implications, and its relationship with other mental health disorders. This understanding enables individuals to make more informed decisions regarding their treatment.

Furthermore, participants underscored an essential need for immersive experiences through visual and audio resources. Visual and audio elements emerged as powerful tools for enhancing user engagement and catering to diverse learning styles and preferences.^52^ Participants enthusiastically endorsed the incorporation of visual resources such as images and videos. Visual elements make them accessible to individuals with varying learning styles. Also, audio resources were highlighted to enhance accessibility and engagement, particularly for those who may not be visual learners. Participants underscored the value of instructional audio in guiding users through immersive sensory experiences, fostering a deeper connection with the content.

### Limitations and future work

This study has several limitations. First, the research was conducted with a small sample size (N=12) of individuals living with PTSD. While this sample size is consistent with prior work on evaluating the acceptability of CAs in the context of mental health,^42–44^ these findings may not be generalizable to the broader population of people with PTSD. Future research should aim to include a larger and more diverse sample, expanding the study to different groups with varied cultural backgrounds and severity of PTSD symptoms.

Second, the scope of interaction with PTSDialogue was limited. The study focused on short-term engagement through interviews, which may not capture long-term usability, effectiveness, or potential issues that could arise over time. Future research should explore long-term studies that involve extended interaction with PTSDialogue over several weeks or months.

Lastly, while this study provides qualitative insights into the acceptance and feasibility of PTSDialogue, it lacks quantitative measures to validate these findings. Incorporating quantitative measures such as usage analytics, participant engagement levels, and clinical outcomes (e.g. reduction in PTSD symptoms) in future studies would strengthen the evidence of the CA efficacy to support self-management for individuals with PTSD. Additionally, future work should aim to compare longitudinal efficacy and acceptance of CA with other forms of digital interventions.

## Conclusion

The prevalence of PTSD as a pervasive health concern underscores the need for effective self-management resources. In response to this challenge, we developed a CA in collaboration with clinical experts to support individuals living with PTSD. In this work, we assess the feasibility and acceptance of PTSDialogue among people living with PTSD. We conducted semi-structured interviews with individuals living with PTSD (N=12) . Our study revealed a high level of acceptance for the CA among participants, highlighting its potential to address critical treatment gaps. Through our findings, we identified key opportunities and challenges associated with integrating CAs into existing healthcare providers and the potential for reducing health inequity and overcoming access barriers. We emphasized the importance of incorporating personalization, privacy-sensitive interactions, and multimedia message delivery in CA design to effectively support this population. We discussed the importance of addressing privacy and ethical concerns to foster trust among users and delved into the human-in-the-loop approach in the context of longitudinal mental healthcare. We have also provided design recommendations aimed at supporting self-management for marginalized populations. Our study contributes to the emerging capabilities of CAs to enhance mental healthcare.
